# Trichotillomania, Trichophagia and Trichobezoar in a Male Paediatric Patient: A Case Report and Literature Review

**DOI:** 10.1016/j.ijscr.2024.109520

**Published:** 2024-03-11

**Authors:** Mansoor Ahmed, Murad Habib, Huma Memon, Rafi Raza Ahmad, Muhammad Amjad Chaudhary

**Affiliations:** Department of Paediatric Surgery, The Children's Hospital, Pakistan Institute of Medical Sciences, Shaheed Zulfiqar Ali Bhutto Medical University, Islamabad, Pakistan

**Keywords:** Trichotillomania, Tricophagia, Trichobezoar, Paediatric, Surgery

## Abstract

**Introduction and importance:**

Trichotillomania and tricophagia, characterized by compulsive hair-pulling and subsequent ingestion which results in a compact mass of hair called trichobezoar. It represents an uncommon psychiatric disorder, especially in young children.

**Case presentation:**

This case report describes a distinctive and rare occurrence of trichotillomania, tricophagia and trichobezoar in a 11-year-old male child. Concerns raised by the parents regarding noticeable hair loss, who initially presented to medical outdoor patient with complaints of abdominal pain on and off from the last one year. He had a history of pica and weight-loss. He was then diagnosed with a gastric trichobezoar for which he was operated upon and a giant trichobezoar was retrieved from his stomach. Post-operatively patient remained admitted in ward and was discharged home on fifth post-operative day and sent for psychiatry evaluation.

**Clinical discussion:**

Trichotillomania and tricophagia often have roots in psychosocial stressors, anxiety, and depression. Children may engage in hair-pulling as a coping mechanism, especially in response to familial or environmental stressors. The literature emphasizes the importance of understanding the psychosocial context to tailor interventions effectively.

**Conclusion:**

Trichotillomania and tricophagia is very rare in paediatric population and if presents a multidisciplinary team comprising of a paediatrition, paediatric surgeon and paediatric psychiatrist should be involved and if diagnosed with a gastric trichobezoar should be removed surgically in order to prevent complications.

## Highlights

• Trichophagia is a disorder which is rare in paediatric age group and rarer in males.
• Trichophagia is a psychiatric disorder and post op psychiatry consultation must be done.
• It should be suspected in patients with scarce hair.
• Multi disciplinary approach needed for the treatment.

## 1 Introduction

Trichotillomania and tricophagia are characterized by the compulsive desire of pulling one's hair with subsequent ingestion, it is infrequently reported in paediatric population [1]. While trichotillomania and tricophagia have been documented in psychiatric literature, the convergence of these behaviors leading to the formation of a trichobezoar requiring surgical intervention is a rare and intriguing phenomenon in the context of early childhood [2] with an incidence of <1 % and is more common in females with simultaneous psychiatric disorders. The overall reported cases of trichotillomania and tricophagia leading to trichobezoar in both genders combined have been <48 since the first reported case in 1968. This case report presents an exceptional instance of these disorders in an 11-year-old male child, further complicated by the development of a trichobezoar necessitating laparotomy for removal [3].

The presentation of trichotillomania and tricophagia in a child of such tender age adds complexity to both diagnosis and management. The intricate interplay of psychological, developmental, and medical factors underscores the importance of a multidisciplinary approach to comprehensively understand and address the unique challenges posed by this case [4]. This report aims to contribute to the limited literature on the co-occurrence of trichotillomania, tricophagia, and trichobezoar in paediatric population, shedding light on the diagnostic intricacies, therapeutic considerations, and the imperative for timely intervention in such exceptional cases [5].

## 2 Case presentation

We report here a rare presentation of an 11-years-old male child from poor socio-economic background who initially presented to medical outdoor patient with complaints of hair fall and abdominal pain on and off from the last one year. He had a history of pica and weight-loss. According to the attendant (mother), patient had history of eating mud since pre school age. Previous history showed normal milestones and no significant stressors in the family. Upon next presentation dermatology consultation was taken by the medicine department for alopecia areata as his temples were getting scarce. The patient presented to surgical outdoor patient with complaints of abdominal pain (epigastric) and on & off vomiting.

**Fig. 1:**
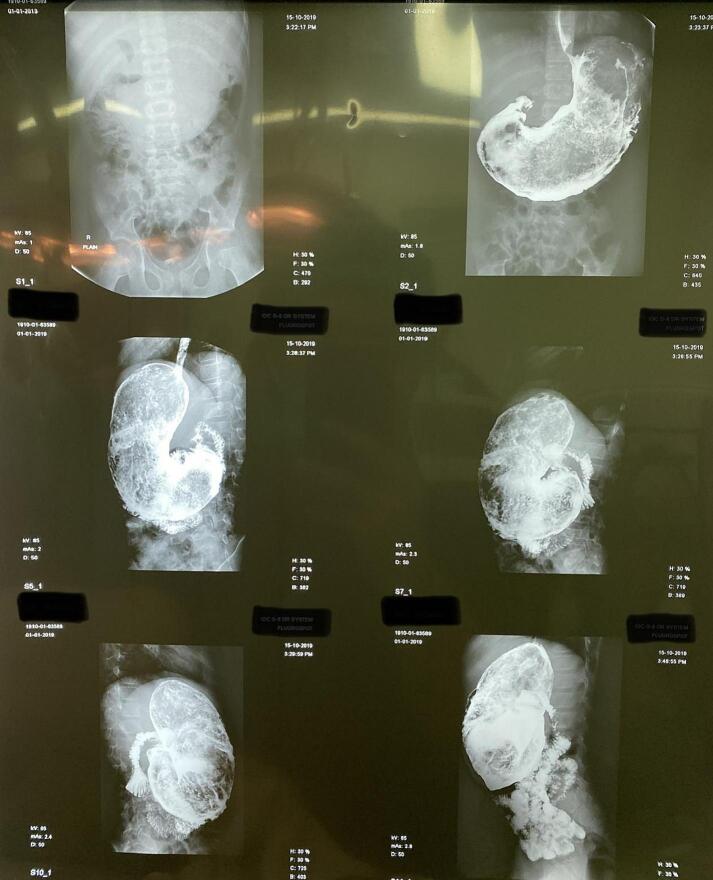
An esophagogram showing gastro trichobezoar.

**Fig. 2:**
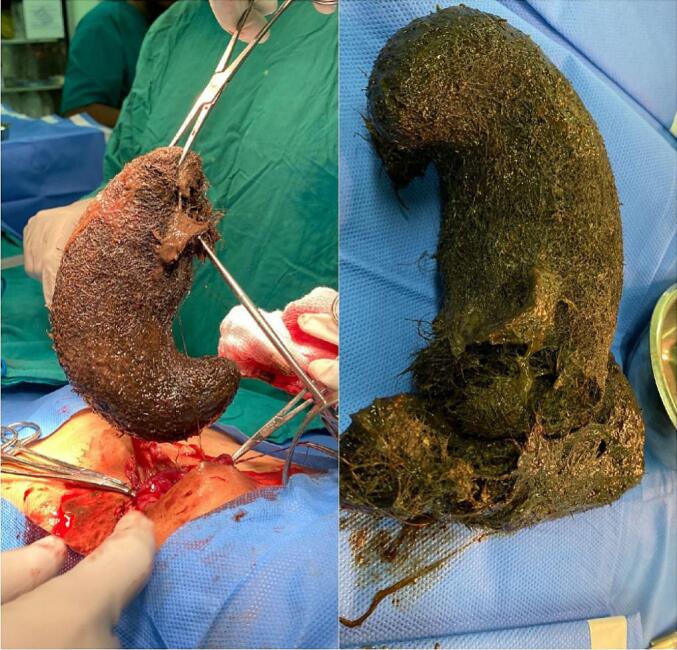
A giant trichobezoar revealed at laparotomy.

On examination, patient was healthy looking with soft abdomen, no tenderness and no distension but feeling of a mass in the epigastrium. Bald patches were observed over the occipital region. Abdominal radiograph was unremarkable whereas ultrasonography of the abdomen revealed a mass in the stomach. Plan was then made to go for barium swallow and hence time was taken for it. An esophagogram revealed a gastric trichobezoar (Fig. 1). Patient was then admitted and planned for surgery. An elective laparotomy was performed through a left upper quadrant transverse incision. Abdomen opened in layers and bulge of bezoar felt in stomach. A gastrotomy was then performed and trichobezoar was removed (Fig. 2). The bezoar was comma shaped hair ball comprosed of hair with mixed gastric contents. Following removal, stomach closed in two layers and abdomen closed in reverse order. Nasogastric tube was passed and patient shifted to in-patient ward. Post-operatively patient remained admitted in ward. Nasogastric tube removed on 3rd post op day and patient allowed oral sips. Patient was then discharged home on fifth post-operative day. Post op period was uneventfull. Patient was then sent for psychiatry evaluation.

## 3 Discussion

Trichotillomania, the compulsive urge to pull out one's hair, and tricophagia, the consumption of the pulled hair, are psychiatric conditions that, when occurr concurrently, can lead to the formation of trichobezoars [6]. Trichotillomania and tricophagia are most commonly observed in females, and their prevalence tends to increase during adolescence and early adulthood. However, the presented case deviates from this pattern, as the patient is an 11-year-old male presented to us with trichotillomania, trichophagia and trichobezoar. The literature suggests that while childhood onset is possible, it is relatively uncommon, making this case noteworthy [7]. Trichotillomania and tricophagia often have roots in psychosocial stressors, anxiety, and depression. Children may engage in hair-pulling as a coping mechanism, especially in response to familial or environmental stressors. The literature emphasizes the importance of understanding the psychosocial context to tailor interventions effectively [8].

Additionally, these behaviors frequently coexist with other psychiatric conditions, such as obsessive-compulsive disorder (OCD), attention-deficit/hyperactivity disorder (ADHD), and anxiety disorders [9]. Diagnosing trichotillomania and tricophagia in children can be challenging due to variations in the presentation of symptoms and the limited ability of young children to articulate their emotional experiences [10]. Various therapeutic modalities have been explored for managing trichotillomania and tricophagia, with cognitive-behavioral therapy (CBT) emerging as a primary intervention. CBT aims to modify negative thought patterns and behaviors, offering a structured and evidence-based approach [11]. Pharmacological interventions, such as selective serotonin reuptake inhibitors (SSRIs), may be considered in severe cases or when comorbidities like anxiety or depression are present. In cases where trichotillomania and tricophagia progress to the formation of trichobezoars, surgical intervention becomes necessary as trichobezoars can lead to gastrointestinal complications, including obstruction, ischemia and perforation [3,12].

The coexistence of trichotillomania, tricophagia and trichobezoar is very rare in this age group [13]. The decision to perform a laparotomy for the removal of the trichobezoar was necessitated by the potential complications associated with this ingested hair mass. The successful surgical intervention in this case highlights the importance of a prompt and accurate diagnosis, as well as the collaborative efforts of psychiatric and surgical specialties in managing such complex presentations [14].

The postoperative course of the patient, marked by an uneventful recovery and subsequent discharge without complications, speaks to the efficacy of the timely intervention and the comprehensive care provided [15]. However, it also prompts consideration of the necessity for ongoing psychiatric and behavioral management to address the underlying trichotillomania and tricophagia. The interdisciplinary collaboration between psychiatrists, pediatricians, and paediatric surgeons is crucial not only during the acute phase but also for long-term management and prevention of recurrence. [16]

The rarity of this case raises questions about the underlying factors contributing to the development of such behaviors in a preadolescent child. Psychosocial stressors, familial dynamics, and potential genetic predispositions may play intricate roles that warrant further investigation [17]. Additionally, the successful outcome of this case invites reflection on the optimal strategies for preventing recurrence and promoting the child's psychological well-being. Further research into the underlying factors and long-term outcomes of such cases is warranted to enhance our understanding and improve the management of these complex psychiatric and surgical presentations in paediatric populations [18] [19].

## 4 Conclusion

Trichotillomania and tricophagia is very rare in paediatric population and if presents a multidisciplinary team comprising of a paediatrition, paediatric surgeon and paediatric psychiatrist should be involved and if diagnosed with a gastric trichobezoar should be removed surgically in order to prevent complications.

## Methodology

Work has been reported in line with the SCARE criteria.

## Informed consent

Patient's attendant (father) provided informed consent prior to study participation.

## Ethical approval

Case reports are exempted from ERB approval in our institution.

## Funding

No funding received for this research.

## Author contribution

Conceptualization: MA.

Data acquisition: HM, RR.

Writing and drafting: MA, MH.

Figures preparation: MH.

Supervision: MAC.

## Guarantor

Mansoor Ahmed.

mansoorahmed1993@live.com

## Registration of research studies

No new drug was used.

## Declaration of competing interest

The authors have no conflicts of interest relevant to this article to disclose.

## Data Availability

The data that support the findings of this study are available from the corresponding author upon request to corresponding author.
